# New Method of Preparing Glass Inlays

**Published:** 1891-03

**Authors:** John Girdwood

**Affiliations:** 65 Queen Street, Edinburgh


### Foreign Correspondence.

### NEW METHOD OF PREPARING GLASS INLAYS.

To the Editor:

In the November number of the International Dental Journal I observe an article by Dr. Bruce, of Melbourne, on the Herbst method of glass inlaying.

Recently, when in America, I had the good fortune to see some beautiful specimens of inlay work, which had been sent over by Dr. Bruce from Germany to a mutual friend in Philadelphia. These were accompanied by a short description that differed in some points from the published one. On my return to Europe, I was induced, by what I had seen in Philadelphia, to try this method, but failed to get anything to please me; but after reading Dr. Bruce's paper in your November number, I renewed my attempts at glass-inlaying and with very satisfactory results. The method I have adopted differs in some details from that of Dr. Bruce and seems simpler and quicker, while the results are equally good. It is chiefly a combination of the methods of Drs. Herbst and Reichter, and is as follows:

The cavity is prepared without undercuts and lined with a piece of No. 60 gold-foil, which is pressed to place by means of small hard balls of cotton-wool; thus a sharp impression of the cavity is got, but if a sharper one be wished a burnisher may be employed, care being taken to anneal often. This is then carefully removed and the resulting matrix about half filled with the glass powder made into a paste with distilled water. The matrix is then held by one corner with a pair of foil-forceps and the glass carefully dried over an ordinary alcohol lamp and fused; more of the paste is added and the operation repeated till the cavity is full. After each firing cracks from shrinkage will be seen in the fused glass, but after the third addition of the paste these will have disappeared. After allowing it to cool, the gold is stripped off and the inlay is ready to set in the tooth. If too full, it can be ground down with fine corundum wheels, followed by Arkansas stones and moose-hide points, with pumice powder and water, which leave the inlay with a fine gloss. I sometimes mix the glass with alcohol instead of distilled water; by its use the powdered glass is less likely to spark when heated.

Those who have adopted the Herbst and Reichter methods will appreciate the saving in time and trouble of non-investment, as well as the further advantage of cutting and polishing the inlay after it is set in the tooth.

JOHN GIRDWOOD, L.D.S., D.D.S.

65 QUEEN STREET, EDINBURGH.

